# Functional Interaction of SCAI with the SWI/SNF Complex for Transcription and Tumor Cell Invasion

**DOI:** 10.1371/journal.pone.0069947

**Published:** 2013-08-02

**Authors:** Camilla Kreßner, Peter Nollau, Robert Grosse, Dominique T. Brandt

**Affiliations:** 1 Institute of Pharmacology, University of Marburg, Marburg, Germany; 2 University Medical Center Hamburg-Eppendorf Institute of Clinical Chemistry, Hamburg, Germany; University of Bristol, United Kingdom

## Abstract

We have recently characterized SCAI (Suppressor of Cancer Cell Invasion), a transcriptional modulator regulating cancer cell motility through suppression of MAL/SRF dependent gene transcription. We show here that SCAI is expressed in a wide range of normal human tissues and its expression is diminished in a large array of primary human breast cancer samples indicating that SCAI expression might be linked to the etiology of human cancer. To establish a functional link between SCAI and tumorigenesis we performed affinity columns to identify SCAI-interacting proteins. Our data show that SCAI interacts with the tumor suppressing SWI/SNF chromatin remodeling complex to promote changes in gene expression and the invasive capacities of human tumor cells. Moreover our data implicate a functional hierarchy between SCAI and BRM, since SCAI function is abrogated in the absence of BRM expression.

## Introduction

Dynamic changes in chromatin architecture are necessary to adapt the transcriptional profile to specific changes of the physiological conditions. The SWI/SNF complex of chromatin-remodeling enzymes uses the energy of ATP-hydrolysis to alter histone-DNA interactions within the nucleosome [1]. The activity of the SWI/SNF chromatin remodeling complex leads to the mobilization of histone octamers along the DNA (nucleosomal sliding) and can thereby promote transcriptional activation or repression of specific genes by facilitating or restricting access of transcription factors and the basal transcriptional machinery to the DNA.

Mammalian SWI/SNF complexes are composed of either BRM or BRG1 and 9–12 additional subunits, which are referred as BRM- or BRG1-associated factors called BAFs [2]. Individual SWI/SNF complexes contain either BRM or BRG1, but not both [3], [4], such that BRM/BAF complexes are structural distinct from BRG1/BAF complexes. Beside their ATPase subunit, the chromatin remodeling complexes differ in the composition of associated cofactors, which stimulate and modulate qualitatively the remodeling activity within the complex [5], [6], [7]. In addition, the associating subunits are believed to mediate targeting of the ATPase subunit to integrate nucleosome remodeling into a physiological context [8].

Mammalian SWI/SNF complexes are involved in the dynamic transcriptional regulation of a large array of genes including cell cycle regulators, signaling proteins, genes regulating the architecture of the cell and adhesion to the extra cellular matrix [9], [10], [11], [12]. In addition, SWI/SNF complexes are critical mediators of RB and p53 to induce cell cycle arrest [13], [14], [15] and are required for BRCA-mediated DNA repair [16], pointing toward a fundamental function of these proteins as tumor suppressors. BRM knockout mice as well as BRG1 heterozygous mice are more prone for cancer development [17], [18], [19]. Analysis of human tumor samples has revealed that BRG1 and BRM are coordinately silenced in various human cancers indicating that silencing of BRG1 and/or BRM could be an important step in the etiology of a significant number and diverse range of human tumors (summarized in [20].

SCAI (suppressor of cancer cell invasion) has been recently characterized as a protein that inhibits the invasive migration of human tumor cells through the control of MAL/SRF signaling [21]. To gain further insight into how SCAI impacts on gene transcription, we have performed a screen for SCAI-interacting proteins. We show here that SCAI is functionally linked to SWI/SNF complexes to promote changes in gene expression that may be critical for tumor cell invasion.

## Materials and Methods

### Reagents, Antibodies and Plasmids

Cell culture reagents were obtained from Invitrogen. α-Flag-agarose (cat. #A2220), HRP-conjugated α-Flag (cat. #A8592), α-HA (cat. #H6533) and α-myc (cat. #A5598) antibodies were from Sigma. Antibodies directed against RhoC (cat. #3430S) and MAPK (cat. #4695) were purchased from Cell signaling, the HDAC-2 antibody was from Abcam (cat. #ab32117) and the α-BRM antibody was purchased from Santa Cruz (cat. #sc17828). The SCAI specific monoclonal antibody 1H2 for immunoblot and the clone 6G6 for immunohistochemistry application has been described before [21]. The pan-cytokeratin antibody (cat. #DLN-09108) and all secondary antibodies were from Dianova. Plasmids containing the cDNA of human BRM as well as an ATPase (K749R) deficient mutant were obtained from Christian Muchard (Institute Pasteur, Paris, France [22]. BRM cDNAs were amplified with specific primers and ligated in pEF-vectors. SCAI plasmids have been described previously [21]. Matrigel was obtained from BD Biosciences (cat. #354230).

### Bacterial Protein Production and Interaction Assays

GST and GST-SCAI (amino acid 35–280) were produced in the Escherichia coli strain DE3 and dialyzed against PBS, 1 mM DTT, 2% glycerol, snap-frozen in liquid nitrogen and stored at −80°C. Association studies were performed as described [21], [23]. A high-salt mouse brain extract was used as a protein source. Bound proteins were eluted by a salt gradient and precipitated with isopropanol. Eluated proteins were separated by SDS-PAGE, stained with colloidal coomassie (Invitrogen) and identified by liquid chromatography tandem mass spectrometry (LC-MSMS) (ZMBH, Heidelberg).

### Cell Based Assays

Human HEK293 and SW13 cells (purchased from ATCC organization) were maintained in DMEM (PAA) supplemented with 10% FBS (GIBCO), 2 mM glutamine, 100 IU/ml penicillin, and 100 mg/ml streptomycin (PAA) at 37°C in a CO_2_ atmosphere. MDA-MB-435 and MDA-MB-231 cells (ATCC organization) were maintained with RPMI (PAA) containing 10% FBS. Cells were transfected using LipofectAMINE 2000 (Invitrogen, cat. #11668) or Ca_2_PO_4_. siRNA was transfected using Matra (IBA-BioTAGnology, cat. # 7-2021-020) or Hiperfect (Qiagen, cat. #3017) according to the instructions of the manufacturer. siRNA-sequences (purchased from IBA) used for this study: SCAI: 5′aa-gtggaatggaacttggtgc-tt; BRM #1∶5′aa-gcccatcgatggtatacat-tt; BRM #2∶5′aa-gaggtgctaagacacttat-tt.

For co-immunoprecipitations HEK293 cells were transfected with the indicated plasmids and cells were lysed after 16 h by sonication in HNG-lysis buffer (25 mM Hepes pH 7.9, 100 mM NaCl, 10% glycerol, 5 mM MgCl_2_, 0.2% NP-40) containing protease (Roche, cat. #05056489001) and phosphatase (Sigma, cat. #P2850/P5726) inhibitor cocktails. Mapping of the SCAI-BRM interaction motif was performed as described above and cells were lysed in RIPA (50 mM Tris pH 7.4, 150 mM NaCl, 2 mM EDTA, 1% Triton X-100, 0,25% DOC, 0,1% SDS). Cleared lysates were incubated with α-Flag-agarose at 4°C for 1 hour with gentle shaking. Immuncomplexes were analyzed by immunoblot using the indicated antibodies.

### Three-dimensional Matrigel Invasion Assays

Matrigel invasion assays were performed as described (Brandt et al 2009). MDA-MB-435 and MDA-MB-231 cells were transfected with specific siRNAs against BRM or SCAI for 48 h. Transwell inserts (Greiner Bio-One) were coated with 50 µl of growth-factor-reduced Matrigel (5 mg/ml). Cells were counted and seeded to the inverted transwell inserts. 1 h later, the lower transwell chamber was filled with 800 µl RPMI1640 containing 0.5% FCS and the upper chamber with 300 µl medium containing 10% FCS. Cells were fixed with 8% FA and visualized with rhodamine-phalloidin and DAPI after 20 h. Invasion assays were analyzed by laser-scanning microscopy and confocal stacks. Numbers of non-invaded versus invaded cells in each optical section from five randomly chosen fields were counted using ImageJ. 3D reconstructions were performed using Zen2010 Software.

### Reporter Gene Assays

HEK 293 cells were transfected with the MAL/SRF reporter 3DA.luc (200 ng), pRLTK (100 ng) and indicated expression plasmids. Cells were maintained in 0.5% FCS media for 16 h until subjected to luciferase measurement. Firefly luciferase activity was normalized to pRLTK (Renilla) luciferase activity.

### Analysis of Protein Expression in Human Tissue

Snap frozen and paraffin-embedded tissue specimens were purchased from US Biomax (Rockville, MD, USA); all donors gave written informed consent and samples were collected under HIPPA approved protocols as stated by the company. None of the authors was involved in collecting samples from participants and ethics committee approval was not necessary. Frozen tissues were pulverized and directly lysed in KLB buffer as previously described [24]. Protein concentrations were determined by the Bradford assay. For Western blot analysis, lysates (20 µg/lane) were separated on 4%–12% SDS-PAGE gradient gels, transferred to PDVF-membranes and probed with the indicated antibodies. Radiographic films were scanned, the integral intensity of each band was determined using the NIH ImageJ software package and signals were normalized to the MAPK signal serving as a reference for equal loading. Tissue sections were deparaffinized by standard protocols and antigen retrieval was performed by boiling in 0.1 M sodium citrate buffer (pH 5.0). After quenching of endogenous peroxidase activity, tissue sections were blocked at 4°C for 2 h in PBS in the presence of 0.2% BSA (Roth), 10% fetal calf serum and 0.3% Triton X-100. Subsequently, slides were incubated overnight at 4°C with the first antibody, washed for 5×5 min each in PBS, incubated with the HRP-labeled secondary antibody for 2 h at 4°C at a dilution of 1∶1000 and stained with 3,3′-diaminobenzidine (Dako).

## Results and Discussion

**Figure 1:**
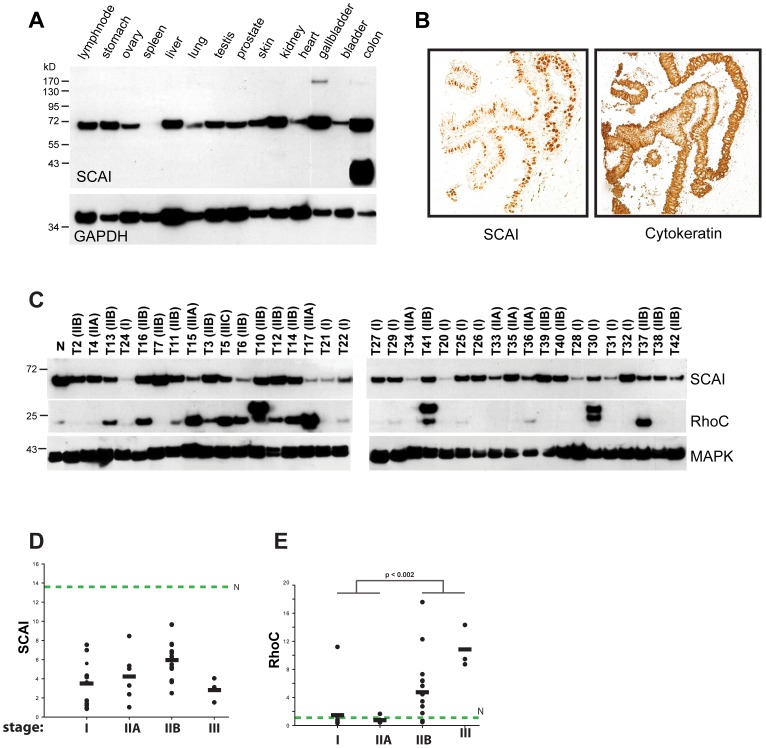
SCAI expression in human tumor samples. (A) Analysis of SCAI expression in human tissue using rat mAB 1H2 (Brandt et al 2009). After stripping, membranes were reprobed with anti-GAPDH mAb serving as a loading control. (B) Breast tissues were deparaffinized and probed with a rat anti-SCAI mAb and a secondary HRP-labeled goat anti-rat antibody. Staining was performed with DAB. To depict cells of epithelial origin, a consecutive tissue section was probed with a pan-specific cytokeratin antibody. (C) Western Blot analysis of SCAI, and RhoC protein expression in breast cancer specimen (n = 36) and normal tissue (N). MAPK served as a loading control. Specimen number and stage of disease according to the AJCC classification are given on top of the figure. (D and E) Relative levels of SCAI and RhoC protein expression in relation to stage of disease. Signal intensities were calculated after densitometric analysis of Western blots shown in (C) normalized to the MAPK signal. Expression levels of SCAI and RhoC in normal breast tissue (N) are given by the green dashed lines. No significant association between tumor stage and levels of protein expression were observed for SCAI whereas levels of RhoC expression were strongly correlated to the stage of disease with higher levels of RhoC in advanced breast cancer.

SCAI is a transcriptional repressor of SRF and a highly conserved protein among vertebrates [21]. The protein expression of SCAI in native human tissues and its sub-cellular localization have not yet been determined. Here, we investigated levels of protein expression of SCAI in a wide range of normal human tissues (Figure 1A). By Western blot analysis we detected expression of SCAI in all normal tissues, except for spleen, with highest levels of expression in colon and gallbladder. Our previous studies on tissue culture cells demonstrated that SCAI is mainly expressed in the nucleus. To determine the subcellular localization of SCAI in native tissue, we stained sections of normal breast tissue. We show that the expression of SCAI is restricted to the duct epithelia of the mammary gland, mainly expressed in the nucleus (Figure 1B). Our initial data revealed that SCAI expression is diminished at the RNA-level in many human malignancies including breast cancer [21]. To further substantiate these findings, we have analyzed a large panel of primary human breast tumors by western blot for SCAI expression. Compared to normal breast tissue, we observed a decrease of SCAI expression in all tumor samples analyzed (Figure 1C), supporting our initial finding that downregulation of SCAI may be linked to tumorigenesis. However, we did not observe a correlation between the breast cancer stage and the reduction of SCAI protein levels (Figure 1 C/D), suggesting that downregulation of SCAI is an early event in tumorigenesis and is not associated with tumor progression. As control for our data set, we analyzed the abundance of RhoC (Figure 1E), and found that enhanced expression of RhoC occurs mainly in late stage tumors, which is in agreement with a publication by Kleer et al. [25].

**Figure 2:**
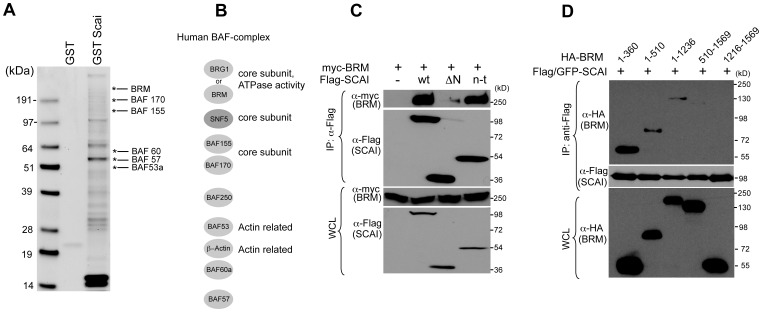
BRM, a core component of the SWI/SNF complex, associates with SCAI. (A) Coomassie stained gel of GST and GST-SCAI (aa 35–280) associated proteins using a mouse brain high salt extract as a source of proteins. (B) Subunit composition of the human SWI/SNF complex (modified from Roberts and Orkin, 2004). (C) SCAI coimmunoprecipitates with the ATPase BRM. HEK 293 cells were transfected with Myc-tagged BRM and full length SCAI, SCAI aa 460–606 (ΔN) and SCAI aa 1–212 (n-t) and subjected to immunoprecipitation using Flag-beads. Immunoprecipitates were analyzed by immunoblot using the indicated antibodies. (D) The N-terminus of BRM is required for SCAI interaction. HEK 293 cells were transfected with Flag/GFP-tagged SCAI and indicated HA-tagged BRM deletion mutants and subjected to immunoprecipitation using Flag-beads. Immunoprecipitates were analyzed by immunoblot using the indicated antibodies.

However, the question of how SCAI impacts on gene regulation at a molecular level as well as the link between SCAI expression and tumor development is at present not fully understood. SCAI does neither share any sequence homologies to other known proteins nor any predicted domain architecture or intrinsic catalytic activity. Therefore, it seemed tempting to speculate that SCAI could serve as an adapter protein that recruits chromatin modifying enzymes, like Histone Deacetylases (HDACs), Methytransferases, or ATP-utilizing chromatin remodeling enzymes to specific genomic regions and thereby controls expression of target genes. To identify the molecular link between SCAI and the dynamic regulation of the chromatin architecture we have performed an affinity screen for SCAI-interacting proteins. A fragment of SCAI comprising amino acids 35–280 was used as bait protein. SCAI-interacting proteins in high salt fraction of mouse brain lysate were separated and analyzed by mass spectroscopy analysis (Figure 2A).

The data showed proteins, mainly involved in histone modifications and having ATPase and DNA helicase activities. Among these, 6 subunits of the SWI/SNF complex associated with SCAI (Figure 2A/B).

We were able to further confirm this potential interaction by co-immunoprecipitation experiments. SCAI and BRM, the central core ATPase subunit of the human SWI/SNF complex, were expressed in HEK 293 cells. SCAI was immunoprecipitated and the precipitates were analyzed for the presence of BRM (Figure 2C). Interestingly, the N-terminal fragment comprising amino acids 1–212 (depicted as n-t), a region that we have previously characterized as a critical region for its biologically activity [21], was sufficient and required for interaction with BRM, whereas a construct lacking the N-terminus (aa 460–606, depicted as deltaN) did not co-immunoprecipitated with BRM. We were also able to map the N-terminal 360 amino acids of BRM as the region required and sufficient to interact with SCAI by co-immunoprecipitation experiments (Figure 2D). However, we have not been able to see association of endogenous BRM and SCAI, indicating that SCAI could be a substoichiometric, non-obligate partner for BRM and that this complex is only operative at certain promoters.

**Figure 3:**
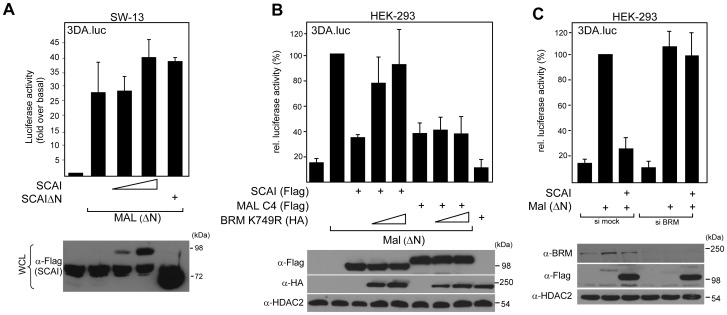
SCAI requires SWI/SNF to modulate SRF-dependent reporter gene activity. SW13 cells were transfected with the SRF reporter 3DA.Luc, pRLTK (Renilla luciferase) and indicated expression plasmids. Reporter gene activity was assessed 48 h after transfection. Statistical analysis of three independent experiments (+/− s.d.) is shown in (A). (B) HEK 293 cells were transfected with the SRF reporter 3DA.Luc, pRLTK and indicated expression plasmids. Reporter gene activity was measured 16 h after transfection. Statistical analysis of three independent experiments (+/− s.d.) is shown. (C) HEK 293 cells were transfected with siRNA specific to hBRM. 48 h later cells were transfected with the SRF reporter 3DA.Luc, pRLTK and an expression plasmid for SCAI wt and the reporter gene activity was measured 16 h later. Statistical analysis of three independent experiments (+/− s.d.) is shown. Representative immunoblots assessing the expression of SCAI/BRM constructs as well as endogenous BRM and HDAC2 as loading control is shown below the bar charts for each experiment. Please note that the Flag-antibody recognizes an unspecific band above 72 kDa in SW13 cells.

Our data further indicate that SCAI requires the presence of a functional SWI/SNF complex to suppress promoter activity. We performed SRF-dependent reporter gene assays in SW13 cells, a human adrenal adenocarcinoma cell line that lacks expression of BRM and the closely related BRG1 protein [26]. Transfection of an active version of the SRF co-activator MAL (MAL ΔN) induced reporter gene activity in these cells, however, unlike to cell lines expressing BRM [21], the co-expression of SCAI did not affect the MAL-induced reporter gene activity in these cells (Figure 3A), indicating that SCAI may be functionally dependent on SWI/SNF-activity to mediate changes in gene expression. We could further show that the expression of an ATPase-deficient mutant of BRM can relieve the inhibition of SCAI on MAL-induced SRF-dependent reporter-activity (Figure 3B). This effect was specific for SCAI, since the repression mediated by a dominant negative version of MAL, a construct that binds to SRF but lacks the transactivation domain [27], was not affected by co-expression of BRM K749R. In addition, we were able to show that siRNA-mediated silencing of BRM abolishes the effect of SCAI on MAL-SRF transcriptional activity (Figure 3C), further supporting our hypothesis of a functional hierarchy between BRM and SCAI. It is at present also not clear whether SCAI can directly modulate the activity of BRM containing SWI/SNF complexes or whether SCAI represents a novel auxiliary factor that mediates recruitment of the complex to specific chromosomal locations.

**Figure 4:**
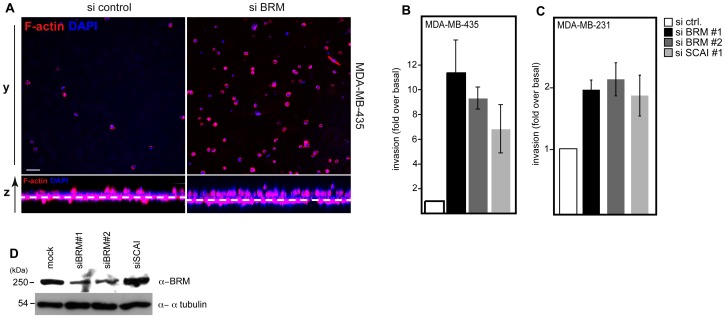
Silencing of the SWI/SNF core subunit BRM phenocopies SCAI mediated effects on invasive cell migration. (A) MDA-MB-435 cells were transfected with indicated siRNAs. After 48 h cells were analyzed for their invasive properties using a 3D matrix (matrigel). Representative images show confocal sections of invaded cells stained for F-actin (red) and DAPI (blue) at 20 µm distance to the transwell membrane. Three-dimensional reconstruction shows a side view of experiments with the location of invaded cells with respect to the transwell membrane (dashed line). A quantification of three independent experiments (+/−s.d.) is shown in (B) for MDA-MB-435 cells and in (C) for MDA-MB-231 cells. (D) MDA-MB-435 cells were processed for immunoblot analysis after 48 h of siRNA treatment and the abundance of BRM protein was assessed using the indicated antibodies.

We next analyzed the functional consequences of this interaction for tumor cell invasion. Our data show that silencing of BRM as also SCAI caused an increase of cell invasion of MDA-MB-435 (Figure 4A/B) as well as MDA-MB-231 (Figure 4C) cells into 3D-matrigel matrices. The moderate effects on cell invasion after siRNA treatment in MDA-MB-231 cells could be explained by the high basal invasion of +/−40%, whereas MDA-MB-435 cells show a basal invasion of +/−5%. These data further supports our hypothesis, that both proteins are functionally linked to each other and may modulate the expression of target genes, which are critical for the invasive behavior of tumor cells.

Taken together, our current data show that SCAI and the SWI/SNF complex interact physically and control gene expression in human cancer cells to regulate invasive cell migration. Our data further indicate that SCAI is functionally dependent on BRM expression, indicating that SWI/SNF could be a downstream mediator for SCAI signaling. The expression analysis of human tumor samples revealed that downregulation of SCAI, like BRM (summarized in [20] is a common phenomenon at least for breast tumors, supporting our previously published data [21]. However, whether diminished expression of SCAI is causatively linked to the development of cancer remains to be resolved in the future. Generation of knock-out mice for SCAI is ongoing and it will be interesting to see whether SCAI depletion can promote spontaneous tumor development in these mice.
